# Effect of different high altitudes on vascular endothelial function in healthy people

**DOI:** 10.1097/MD.0000000000019292

**Published:** 2020-03-13

**Authors:** Ning Fan, Cun Liu, Ming Ren

**Affiliations:** a Graduate School of Qinghai University; b Qinghai Cardiovascular Hospital; c The Affiliated Hospital of Qing Hai University, Xi Ning, Qing Hai, China.

**Keywords:** rapid entrance into different altitudes, vascular endothelial growth factor, nitric oxide, asymmetric dimethylarginine, hypoxia-inducible factor 1, endothelin-1

## Abstract

**Background:**

The aim of the study was to provide a theoretical basis for the early diagnosis and prediction of acute altitude sickness, to provide a better entry mode for healthy people from plain areas to plateau areas, and to preliminarily clarify the possible mechanism of this approach.

**Methods:**

We measured endothelin-1 (ET-1), asymmetric dimethylarginine (ADMA), vascular endothelial growth factor (VEGF), nitric oxide (NO), and hypoxia-inducible factor 1 (HIF-1) levels in each sample and determined flow-mediated dilation (FMD) values using a portable OMRON color Doppler with a 7.0- to 12.0-MHz linear array probe. We used the Lewis Lake score to diagnose acute mountain sickness (AMS) and to stratify the disease severity.

**Results:**

We found no cases of AMS at any of the studied elevation gradients. We found significant differences in FMD values between individuals when at 400 m above sea level and when at 2200, 3200, and 4200 m above sea level (*P* < .05) but found no significant differences among those at 2200, 3200, and 4200 m. Our variance analysis showed that serum ET-1, VEGF, ADMA, NO, and HIF-1 levels in individuals at ≥3000 m and those at subplateau and plain areas (<3000 m) significantly differed (*P* < .05). The level of these factors also significantly differed between individuals at elevation gradients of plateau areas (3260 m vs 4270 m) (*P* < .05). We found no significant differences in serum ET-1, VEGF, and ADMA levels between individuals at the plateau (2260 m) and plain (400 m) areas (*P* > .05). NO and HIF-1 levels were significantly different in serum samples from individuals between the plateau (2260 m) and plain (400 m) areas (*P* < .05). However, with increasing altitude, the NO level gradually increased, whereas ET-1, ADMA, VEGF, and HIF-1 levels showed a decreasing trend. With the increase of altitude, there is no correlation between the trend of FMD and hematologic-related factors such as VEGF, NO, and HIF-1.

**Conclusion:**

A healthy young male population ascending to a high-altitude area experiences a low incidence of AMS. Entering an acute plateau exposure environment from different altitude gradients may weaken the effect of acute highland exposure on vascular endothelial dysfunction in healthy individuals. Changes in serum ET-1, VEGF, ADMA, NO, and HIF-1 levels in healthy young men may be related to the body's self-regulation and protect healthy individuals from AMS. A short stay in a subplateau region may initiate an oxygen-free preconditioning process in healthy individuals, thereby protecting them from AMS. Noninvasive brachial artery endothelial function test instead of the detection of invasive hematologic-related factors for early diagnosis and prediction of the occurrence and severity of acute high-altitude disease is still lack of sufficient theoretical basis.

## 1 Introduction

Acute mountain sickness (AMS) occurs in individuals who quickly ascend from a plains or low-altitude area to a high-altitude plateau. Symptoms begin within hours to days after the high-altitude exposure with various clinical syndromes. The incidence of AMS is high, and the threat to life is serious. The syndrome includes acute mild altitude disease, high-altitude pulmonary edema (HAPE), and high-altitude brain edema.^[1]^ The economic and social development in plateau areas worldwide has resulted in the exposure of an increasing number of individuals to rapid high-altitude changes. Its pathogenesis mechanisms are not completely understood. In addition, the diagnosis and prevention of AMS are urgent issues for the plateau health industry. The atmospheric pressure and partial pressure of oxygen in the atmosphere decrease as the altitude increases, resulting in an anoxic environment that threatens the health of individuals entering plateaus. Hypoxia is the 1st environmental factor that affects the body when entering a high-altitude area and has the most serious impact on health. The hypoxic environment alters vascular endothelial cell function and causes an imbalance in various regulatory factors. High-altitude hypoxic environments may promote the synthesis and release of nitric oxide (NO)^[2,3]^ and prostacyclin (epoprostenol, PGI2)^[4]^ and increases in endothelin-1 (ET-1),^[5,6]^ thromboxane A2,^[7]^ and inflammatory factors.^[8]^ Flow-mediated dilation (FMD) values decrease as the altitude increases.^[9]^ These changes lead to increased vascular permeability and decreased antioxidant capacity, and high-altitude pulmonary hypertension, HAPE, and high-altitude cerebral edema may ensue.^[10]^ Therefore, changes in vascular endothelial cell function may act as an important early warning sign and diagnostic index and play an important role in the occurrence and development of AMS. However, the related pathogenetic mechanisms remain unclear, and medical professionals lack effective diagnostic and predictive indicators. In recent years, there has been extensive research on healthy individuals entering a plateau vertically from the plain areas; however, there are few studies on individuals entering a plateau by gradients of elevation. We determined FMD values, NO, ET-1, asymmetric dimethylarginine (ADMA), vascular endothelial growth factor (VEGF), and hypoxia-inducible factor 1 (HIF-1) levels in healthy individuals who entered a plateau area from the plain area by gradients of elevation to assess the mechanism of the occurrence and development of AMS, identify predictive markers for the early diagnosis and prognosis of acute altitude sickness, and determine an optimal entry mode for healthy individuals into plateau areas from the plain areas.

## 2 Methods

These recruits are all soldiers serving in the military service. They are all nationally arranged to participate in the construction of plateau and frontier areas in Qinghai. Their ages are between 20 and 30 years old. According to the organizational arrangements, they staggered at 400 m (convening point), 2260 m, and 3260 m above sea level for a short stay, and eventually reached 4270 m above sea level. The final place of the military service is to carry out the work of building the motherland.

We collected samples from 48 male volunteers from a plain area (400-m elevation) who entered the cities of Xining (2260 m), Zeku County (3260 m), and Maxin County (4270 m). We collected blood samples from these individuals within the first 24 hours of their arrival at the high-altitude area and 48 hours after that. We centrifuged samples at 3000 rpm for 10 minutes and collected the serum in aseptic dry freezing tubes and stored them at −80°C. We used ELISA kits (Shanghai Dan Shi Biotechnology, Shanghai, China) to measure serum NO, VEGF, ET-1, ADMA, and HIF-1 levels at the same time.

We used an OMRON portable color Doppler ultrasound instrument with a 7.0- to 12.0-MHz linear array probe to determine FMD. The vascular endothelial diastolic function of the brachial artery was calculated by detecting the diastolic response of the brachial artery to reactive hyperemia (blood flow mediated endothelium-dependent vasodilatation). The individuals rested for 10 to 15 minutes before the examination, and for the examination, they were in a supine position, with the right arm abducted and stretched out and the palm upwards. We scanned the brachial artery using 2-dimensional ultrasound at 2 to 10 cm from the elbow and then the long axis of the brachial artery. The end diastolic diameter was measured, sound image was clear, and gain, depth, and other conditions were fixed. We marked the probe position to ensure consistency in subsequent measurements. We measured the base diameter of the brachial artery (*D*1) at the peak of the R wave at the time of simultaneous recording. We placed the cuff of the sphygmomanometer at the elbow, and we pressurized the air to 280 mm Hg (1 mm Hg = 01133 kPa). We recorded the brachial artery internal diameter (*D*2) 60 to 90 seconds after 4 to 6 minutes, measuring 3 cardiac cycles each time and calculating the average value. We used *V*% = [(*D*2 – *D*1)/*D*1] × 100% to calculate the rate of change in blood vessel diameter. We recorded the blood flow velocity, flow volume, resistance index, and heart rates before and after brachial artery compression.

We used SPSS 22.0 statistical software to process all data. All normally distributed data were expressed as the mean ± standard deviation (*x* ± *s*). We used variance analysis to compare FMD values and blood-related factors at different altitudes in healthy individuals. Pearson correlation was used to determine whether there was a correlation between FMD and blood-related factors with elevation. We used a test level *α* = 0.05.

All subjects signed an informed consent. The study was approved by the ethics committee of the Affiliated Hospital of Qinghai University, and the individual data and examination results of the subjects were kept strictly confidential and kept in the laboratory of the cardiovascular department of the Department of Cardiology.

## 3 Results

We found no cases of AMS at any of the elevation gradients studied. We found significant differences in FMD values between individuals when at 400 m above sea level and when at 2200, 3200, and 4200 m above sea level (*P* < .05) but found no significant differences among those at 2200, 3200, and 4200 m (Table 1). Our variance analysis showed that serum ET-1, VEGF, ADMA, NO, and HIF-1 levels in individuals at ≥3000 m and those at subplateau and plains areas (<3000 m) significantly differed (*P* < .05), and they significantly differed between individuals at elevation gradients of plateau areas (3260 m vs 4270 m) (*P* < .05). We found no significant differences in serum ET-1, VEGF, and ADMA levels between individuals at plateau (2260 m) and plains (400 m) areas (*P* > .05). NO and HIF-1 levels were significantly different in serum samples from individuals between plateau (2260 m) and plains (400 m) areas (*P* < .05). However, as the altitude increased, the NO level gradually increased, whereas ET-1, ADMA, VEGF, and HIF-1 levels showed a decreasing trend (Table 2). With the increase of altitude, there is no correlation between the trend of FMD and hematologic-related factors such as VEGF, NO, and HIF-1 (Table 3).

**Table 1:**
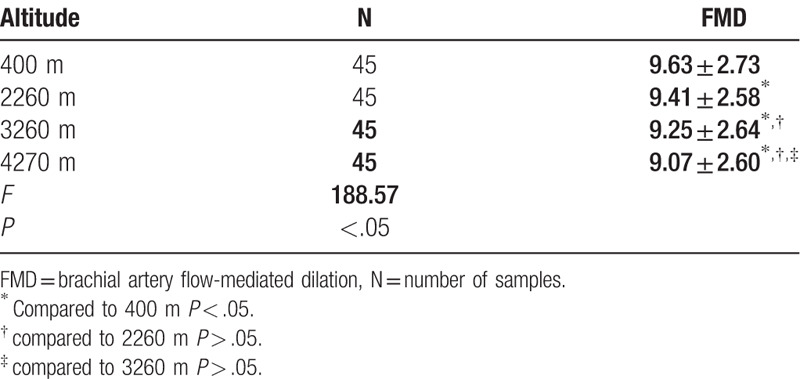
Results of variance analysis of 45 healthy individuals at different altitude FMD levels (*x* ± *s*).

**Table 2:**
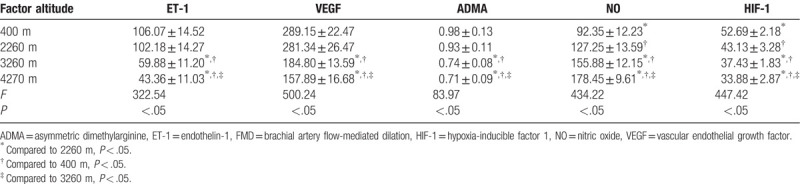
Results of variance analysis of EF-1, VEGF, ADMA, NO, and HIF-1 levels from 45 healthy individuals at different elevations 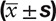.

**Table 3:**
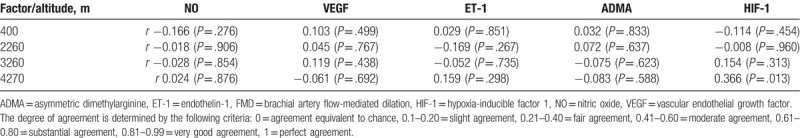
The correlation between FMD level and NO, ET-1, VEGF, ADMA, and HIF-1 at different altitudes.

## 4 Discussion

The pathogenesis of AMS is complicated. Most studies have reported an association between the occurrence of AMS and oxidative stress reactions.^[11–13]^ Our results indicated that as the altitude increased, the FMD values decreased. Serum ET-1, ADMA, HIF-1, and VEGF levels decreased, whereas the NO level increased as the altitude increased. In addition, none of our healthy male volunteers experienced AMS, which may be because they gradually ascended, thereby reducing the occurrence of AMS. Individuals who enter high-altitude areas from low-altitude areas have long experienced AMS at a high rate, which restricted the development of plateau societies. According to the Lewis Lake diagnostic criteria, the incidence of AMS is 0% at 2500 to 3000 m and 10% at approximately 3000 to 4000 m.^[14]^ Karinen et al reported that the incidence of AMS was 10% at 3500 m and 21% at 4300 m.^[15]^ However, the incidence of AMS is influenced by age, sex, genetic background, and the manner and speed of ascent.^[16]^ Therefore, the incidence may differ among different experimental groups. In 2010, Ren et al reported an incidence of AMS of 57.2%, which was calculated by conducting a large-sample epidemiologic survey in China using data from individuals who took a plane from the plains to Lhasa (3600 m). They found that 12.07% of the subjects required hospitalization, and headache was the 2nd most frequent symptom in 74.98% of those affected.^[17]^ Studies have shown that low-pressure hypoxia induces endothelial dysfunction and promotes oxidative stress reactions,^[18,19]^ thereby altering physiologic processes. However, the association between endothelial function and AMS is unclear.

The FMD was the 1st method developed to evaluate endothelial function via noninvasive brachial artery ultrasound.^[20]^ Boos et al found that compared with individuals at a low altitude, those at a high altitude had narrower vascular diameters, lower blood flow, and lower FMD values.^[9]^ Our results showed that the FMD values of subjects did not significantly decrease after acute altitude exposure (2200 m) in the subplateau and plateau groups (3200 and 4200 m), suggesting that entering the plateau area from gradients of altitude can reduce damage to vascular endothelial function under hypoxic conditions.

The NO is the strongest known blood vessel dilator. It protects the body from acute altitude sickness and reduces its symptoms. Altundag et al^[21]^ compared NO values at sea level and at high altitude to investigate the effect of high altitude on NO levels. The mean NO measurement was 74.2 ± 41 parts-per-billion (ppb) at high altitude and 93.4 ± 45 ppb at sea level, and this change in NO depending on the altitude was statistically significant (*P* < .001). The NO level decreases at high altitude even under favorable weather conditions, regarding factors such as temperature, humidity, and wind. A Chinese study has shown that during the early stage of high altitude exposure, ET release in the mouse brain significantly increases, and the NO/ET ratio decreases and then drops sharply at high-altitude areas. Further, NO release was found to increase 10 days after entering the plateau, and the NO/ET ratio increased. On day 13 after entering the plateau, the release of NO began to decrease. Our study showed that NO level did not significantly decrease but rather increased. We believe that with altitude changes from plains to plateau areas, vascular-protective NO release may occur because of the body's self-regulation, which can reduce damage due to acute hypoxia and improve vascular endothelial function, thereby protecting the body from AMS.

An experimental study found that serum ET-1 levels in individuals arriving quickly at a plateau from the plains or low altitudes were significantly increased.^[22]^ The increase in ET-1 is considered a pathogenetic mechanism of hypoxic pulmonary hypertension,^[23,24]^ and it plays a role in the development of AMS. Ceolotto et al^[25]^ found that blood ET-1 concentration is directly related to pulmonary artery pressure. NO inhibits ET-1 expression and synthesis at the gene level.^[26]^ Comellas et al found that serum ET-1 level was positively correlated with Pulmonary artery Pressure in athletes.^[27]^ They observed that after acute altitude exposure at gradients of altitude, the blood ET-1 level of subjects was affected by altitude, and the concentration did not significantly increase but showed a decreasing trend, suggesting that vascular endothelial function was not seriously damaged. Our study found that serum ET-1 levels did not significantly increase and showed a decreasing trend with gradients of altitude. We speculate that with a gradient increase in elevation from plains to plateau areas, an increased NO level inhibits the synthesis and expression of *ET-1*, which can reduce damage to vascular endothelial function due to acute hypoxia and protect the body from AMS.

The body is in a state of hypoxia. In vivo, with HIF-1a activation, HIF-1a can increase VEGF expression through gene regulation, making the body produce more VEGF.^[28]^ After VEGF binds to its receptor, the endothelial cell structure changes, and vascular permeability increases, which eventually causes tissue edema and induces AMS. Currently, the mechanism of VEGF expression in the pathogenesis of AMS is understood as follows: HIF-1a initiates VEGF transcription under hypoxia and makes VEGF RNA more stable and hypoxia increases VEGF RNA and adenosine levels, and high VEGF contents in patients with AMS at high altitudes are correlated with the severity of the syndrome.^[29,30]^ These results suggest that VEGF is an important pathogenic factor of AMS. However, Chinese studies have shown that HIF-1 alpha mRNA expression levels in rats were higher in a high-altitude group than in a low-altitude control group; however, they were lower than those in the moderate elevation group and showed a certain trend of change; that is, on the 1st day, the HIF-1 alpha mRNA expression level obviously increased and then decreased and remained stable. These studies found that acute hypoxia could induce high HIF-1 alpha expression, and chronic hypoxia can downregulate the TERT-1 alpha gene and protein expression levels.^[31,32]^ A study in a foreign country^[33]^ showed that serum VEGF levels in high-altitude areas increased in contrast to those in plains areas; however, there was no significant effect on serum VEGF levels in subjects after a rapid advance of 3650 m to a high elevation of 5200 m after acclimatization. In this study, healthy individuals arrived at the high-altitude area without AMS, and their serum VEGF concentrations decreased, suggesting that vascular endothelial function was not seriously altered, which indicates that a gradual ascent to the high-altitude area alleviated the effects on vascular endothelial function and reduced the occurrence of AMS. Our study found that serum VEGF and HIF-1 levels were not significantly increased and showed a decreasing trend with gradients of altitude. We speculate that HIF-1 factor expression at low levels leads to the low VEGF expression level with increases in altitude, thereby reducing the damage to vascular endothelial function by systolic factors and eliminating acute altitude sickness.

The ADMA is an endogenous inhibitor of NO synthase and can effectively inhibit NO generation.^[34,35]^ Studies have confirmed that ADMA is a predictor of endothelial dysfunction and that ADMA is also closely related to various pulmonary vascular diseases, and its mechanism involves several pathophysiologic processes, such as Pulmonary arterial hypertension, lung vascular endothelial injury, and pulmonary fibrosis.^[36,37]^ However, the role of ADMA in the pathogenesis of AMS is unclear. Our study found that 45 healthy individuals who were transported from a plains area to subplateau area at 2260 m and, 2 days later, from there to a plateau area above 3000 m experienced no AMS. At the same time, serum ADMA levels did not increase but instead showed a decreasing trend. We believe that through gradients of elevation from a plains to a plateau area, ADMA expression can be reduced, thereby decreasing NOS inhibition and increasing NO expression, which can reduce damage to vascular endothelial function due to acute hypoxia and protect the body from AMS.

Our study shows that there is no significant correlation between FMD and serum NO, VEGF, and HIF-1 with the increase of altitude. The analysis may be due to the different characteristics of the subjects when they enter the plateau, such as tools, altitude, speed of entering the plateau, basic diseases of the subjects, inheritance factors, and so on. It may also be due to the fact that we collect fewer samples and do not advance. Long-term dynamic observation of the changes of various indicators in the subjects, so we cannot draw a conclusion that the noninvasive brachial artery endothelial function test can replace the detection of the content of invasive hematologic-related factors to early diagnose and predict the occurrence and severity of acute high-altitude disease.

Studies on mountaineers have confirmed that when mountain climbers enter high-altitude areas before hypoxia, preconditioning effectively reduces AMS.^[38]^ A Chinese study on soldiers has shown that those ascending to a plateau gradually experience less AMS. Hypoxia preconditioning before entering a plateau can significantly reduce the occurrence of AMS and its symptoms. Individuals arriving at a plateau area directly from the plains are affected by AMS at incidences of 30% to 80%. Although the occurrence of AMS can be reduced by a gradual ascent, approximately 30% of individuals continue to experience acute altitude sickness. However, 16% to 30% of individuals who ascend straight to the plateau do not develop AMS, which suggests genetic susceptibilities to AMS.^[14]^

In conclusion, after hypoxic preconditioning at lower altitudes, the incidence of AMS was significantly reduced. We found no significant decrease in vascular endothelial function (FMD) in healthy individuals. ET-1, VEGF, ADMA, and HIF-1 levels in the venous blood of healthy individuals did not significantly increase and the NO level did not significantly decrease with altitude elevation. Our results suggest that changes in NO, ET-1, VEGF, ADMA, and HIF-1 levels, and FMD values protect the body from AMS. We speculate that the following are protective mechanisms against AMS: Through gradients of elevation from plains to plateau areas, ADMA expression decreases, thereby reducing NOS inhibition and increasing NO expression. A high NO level inhibits the synthesis and expression of *ET-1*, which can reduce damage to vascular endothelial function due to acute hypoxia and protect the body from AMS. A low HIF-1 factor expression level inhibits VEGF expression, thereby reducing damage to vascular endothelial function and eliminating acute altitude sickness. Yet unknown genetic differences. High-altitude disease may be prevented by hypoxia tolerance genes, which protect the body from altitude sickness. After the establishment of the Cacao National Natural World Natural Heritage Park (with an average altitude of >4000 m), the incidence of AMS in visitors was reduced by recommending a short stay in a subplateau region (such as Xining, 2260 m) for anoxia preconditioning before ascending to the plateau. There are still some differences between our results and those of other studies. Analyzing the reasons, we speculate that it may also be related to the genetic background of the population, undetected basic diseases, the speed of entering the plateau, the altitude of entering the plateau, the time of staying at different altitudes, and the time of collecting blood samples. In addition, the population included in this experiment is small. To solve this problem, on the one hand, large-scale field experiments are needed. On the other hand, it is necessary to track the factor level of this group further after a long period of high-altitude exposure. In addition, no acute altitude sickness happened in the population included in this experiment, and the changes of serologic factors mentioned above in vivo during the occurrence of acute altitude sickness could not be observed. Therefore, further field experiments and rationalization are needed to use NO, VEGF, and HIF-1 as predictors of early diagnosis and severity of acute altitude sickness. However, we put forward a better entry mode for healthy people to rush into the plateau earlier, and preliminarily speculated on its possible mechanism. We believe that the above research will bring certain guiding significance for social production practice and tourism in plateau area.

## 5 Conclusion

A healthy young male population ascending to a high-altitude area experiences a low incidence of AMS. Entering an acute plateau exposure environment from different altitude gradients may weaken the effect of acute highland exposure on vascular endothelial dysfunction in healthy individuals. Changes in serum ET-1, VEGF, ADMA, NO, and HIF-1 levels in healthy young men may be related to the body's self-regulation and protect healthy individuals from AMS. A short stay in a subplateau region may initiate an oxygen-free preconditioning process in healthy individuals, thereby protecting them from AMS. Noninvasive brachial artery endothelial function test instead of the detection of invasive hematologic-related factors for early diagnosis and prediction of the occurrence and severity of acute high-altitude disease is still lack of sufficient theoretical basis.

## Acknowledgment

The authors thank Enago (www.enago.cn) for its linguistic assistance during the preparation of this manuscript.

## Author contributions

**Data curation:** Ning Fan, Cun Liu, Ming Ren.

**Formal analysis:** Cun Liu, Ming Ren.

**Funding acquisition:** Ming Ren.

**Writing – original draft:** Ning Fan.
